# A rare case of sporadic multiple trichoepitheliomas

**DOI:** 10.11604/pamj.2015.21.19.6845

**Published:** 2015-05-08

**Authors:** Krich Sanaa, Mernissi Fatima Zahra

**Affiliations:** 1 Dermatological Department, Hassan II University Hospital, Fes, Morocco

**Keywords:** Trichoepithelioma, basal cell carcinoma, Morocco

## Image in medicine

A 60-year-old male reported with multiple growths facial tumors on the face and scalp for 44 years around her puberty. On examination multiple firm translucent papules and nodules on the face (A) especially in the nasolabial fold, the medial part of the eyebrows and preauricular area (B) and in the scalp (C) with varying sizes from 5 mm to 2 cm. Dermoscopy was objectived telangectasic vessel at the periphery of lesions (D). No other contributory medical history or similar cases in the family were reported by the patient. With the above features a clinical differential diagnosis of multiple basal cell carcinoma (BCC), multiples trichoepitheliomas or Bourneville's tuberous sclerosis was considered. Histopathological examination of the lesions was in favor of nodular BCC then another skin biopsy objectified Horn cysts characteristics of trichoepithelioma. The diagnosis of multiple Trichoepitheliomas was taken and laser ablation was planned. Trichoepithelioma is a rare benign skin lesion that originates from hair follicles. It usually appears as multiple lesions in the autosomal dominant type or as an individual flesh colored papule or nodule measuring 2 to 8 mm in the sporadic type. Histologically, it is similar to BCC, and although benign, with a rare risk of malignant transformation. Solitary lesions can be excised. In the case of multiple tumors, surgical approach may not be feasible. Other treatments have been proposed in the literature (dermabrasion, electro-surgery and laser surgery…), but the results of these procedures vary.

**Figure 1:**
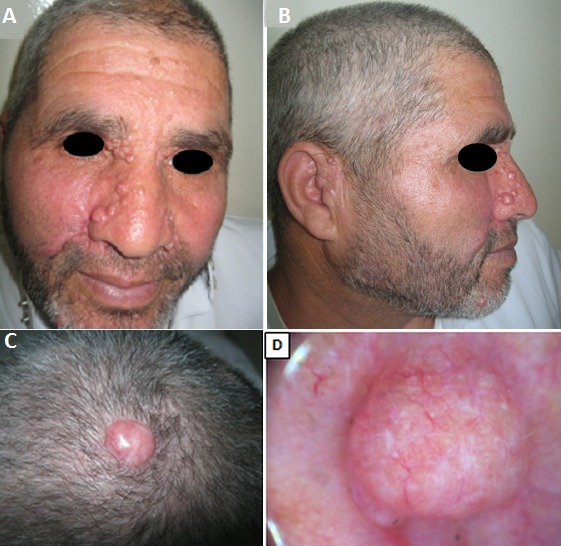
A) multiple firm translucent papules and nodules on the face; B) translucent papules and nodules in the nasolabial fold, the medial part of the eyebrows and preauricular area; C) translucent papule on the scalp; D) telangectasic vessel at the periphery of lesions on dermoscopy

